# The Nonsteroidal Anti-Inflammatory Drug Ketorolac Alters the Small Intestinal Microbiota and Bile Acids Without Inducing Intestinal Damage or Delaying Peristalsis in the Rat

**DOI:** 10.3389/fphar.2021.664177

**Published:** 2021-06-04

**Authors:** Barbara Hutka, Bernadette Lázár, András S. Tóth, Bence Ágg, Szilvia B. László, Nóra Makra, Balázs Ligeti, Bálint Scheich, Kornél Király, Mahmoud Al-Khrasani, Dóra Szabó, Péter Ferdinandy, Klára Gyires, Zoltán S. Zádori

**Affiliations:** ^1^ Department of Pharmacology and Pharmacotherapy, Semmelweis University, Budapest, Hungary; ^2^ Pharmahungary Group, Szeged, Hungary; ^3^ Department of Medical Microbiology, Semmelweis University, Budapest, Hungary; ^4^ Faculty of Information Technology and Bionics, Pázmány Péter Catholic University, Budapest, Hungary; ^5^ 1st Department of Pathology and Experimental Cancer Research, Semmelweis University, Budapest, Hungary

**Keywords:** ketorolac, nonsteroidal anti-inflammatory drug, enteropathy, microbiota, bile acid, peristalsis, hyodeoxycholic acid

## Abstract

**Background:** Nonsteroidal anti-inflammatory drugs (NSAIDs) induce significant damage to the small intestine, which is accompanied by changes in intestinal bacteria (dysbiosis) and bile acids. However, it is still a question of debate whether besides mucosal inflammation also other factors, such as direct antibacterial effects or delayed peristalsis, contribute to NSAID-induced dysbiosis. Here we aimed to assess whether ketorolac, an NSAID lacking direct effects on gut bacteria, has any significant impact on intestinal microbiota and bile acids in the absence of mucosal inflammation. We also addressed the possibility that ketorolac-induced bacterial and bile acid alterations are due to a delay in gastrointestinal (GI) transit.

**Methods:** Vehicle or ketorolac (1, 3 and 10 mg/kg) were given to rats by oral gavage once daily for four weeks, and the severity of mucosal inflammation was evaluated macroscopically, histologically, and by measuring the levels of inflammatory proteins and claudin-1 in the distal jejunal tissue. The luminal amount of bile acids was measured by liquid chromatography-tandem mass spectrometry, whereas the composition of microbiota by sequencing of bacterial 16S rRNA. GI transit was assessed by the charcoal meal method.

**Results:** Ketorolac up to 3 mg/kg did not cause any signs of mucosal damage to the small intestine. However, 3 mg/kg of ketorolac induced dysbiosis, which was characterized by a loss of families belonging to Firmicutes (*Paenibacillaceae*, *Clostridiales Family XIII*, *Christensenellaceae*) and bloom of *Enterobacteriaceae*. Ketorolac also changed the composition of small intestinal bile by decreasing the concentration of conjugated bile acids and by increasing the amount of hyodeoxycholic acid (HDCA). The level of conjugated bile acids correlated negatively with the abundance of *Erysipelotrichaceae*, *Ruminococcaceae*, *Clostridiaceae 1*, *Muribaculaceae*, *Bacteroidaceae*, *Burkholderiaceae* and *Bifidobacteriaceae*. Ketorolac, under the present experimental conditions, did not change the GI transit.

**Conclusion:** This is the first demonstration that low-dose ketorolac disturbed the delicate balance between small intestinal bacteria and bile acids, despite having no significant effect on intestinal mucosal integrity and peristalsis. Other, yet unidentified, factors may contribute to ketorolac-induced dysbiosis and bile dysmetabolism.

## Introduction

Nonsteroidal anti-inflammatory drugs (NSAIDs) are among the most commonly used prescription and over-the-counter medicines (Lanas and Scarpignato, 2006). They are widely used to treat inflammatory pain and reduce fever, but their use is associated with significant gastrointestinal (GI) adverse events. Besides having the potential to seriously damage the stomach and duodenum, NSAIDs can also damage the small intestine. NSAID enteropathy can occur in up to 70% of chronic NSAID users and may result in a wide variety of complications, ranging from mucosal inflammation and protein loss to ulcers, and even perforations (Maiden et al., 2005). The pathogenesis of enteropathy is complex and still insufficiently understood. It likely involves multiple mechanisms, including topical damaging effects of NSAIDs, inhibition of cyclooxygenase (COX)-mediated prostaglandin (PG) synthesis, and increased toxicity of luminal aggressive factors, like intestinal bacteria and bile acids (Wallace, 2013; Takeuchi and Satoh, 2015; Bjarnason et al., 2018).

The importance of bacteria in NSAID enteropathy has been demonstrated repeatedly by numerous studies. Antibiotic-treated or germ-free animals are largely protected against NSAID-induced small bowel damage (Kent et al., 1969; Robert and Asano, 1977; Uejima et al., 1996). Bacteria may aggravate mucosal damage by several ways, including delayed ulcer healing (Elliott et al., 1998) and increased deconjugation and enterohepatic recirculation of NSAIDs (LoGuidice et al., 2012). It has also long been known that NSAIDs cause marked alterations in the composition of intestinal microbiota (Kent et al., 1969; Dalby et al., 2006; Craven et al., 2012; Blackler et al., 2015; Liang et al., 2015; Rogers and Aronoff, 2016; Maseda et al., 2019). This dysbiosis is typically characterized by a dramatic shift from Gram-positive to Gram-negative organisms, and may exacerbate NSAID-induced mucosal damage as well. Indeed, the abundance of Gram-negative bacteria showed positive correlation with the severity of mucosal injury (Blackler et al., 2015).

Although NSAID-induced dysbiosis is well documented, little is known about the underlying mechanisms. Because inflammation-related perturbations in the microenvironment favor the growth of Gram-negatives, such as *Enterobacteriaceae* (Zeng et al., 2017), NSAID-induced mucosal injury is likely to be a major determinant of dysbiosis. This resonates well with studies showing that microbial alterations appeared in rodents with moderate to severe, but not with minimal or mild enteropathy (Reuter et al., 1997; Craven et al., 2012). In addition, the composition of microbiota changed with time and disease progression (Maseda et al., 2019). However, there is some evidence that certain NSAIDs can induce dysbiosis without any detectable intestinal damage as well (Uejima et al., 1996; Lu et al., 2018), suggesting that other factors than mucosal inflammation may also contribute to NSAID-induced microbial alterations.

Several NSAIDs, such as ibuprofen, naproxen, diclofenac or celecoxib have been reported to possess direct antimicrobial properties *in vitro* (Jiménez-Serna and Hernández-Sánchez, 2011; Thangamani et al., 2015; Chan et al., 2017). It remains unclear whether these effects have any significant relevance in terms of dysbiosis, but it is noteworthy that ibuprofen and celecoxib are active mainly against Gram-positive bacteria *in vitro* (Jiménez-Serna and Hernández-Sánchez, 2011; Obad et al., 2015; Thangamani et al., 2015; Chan et al., 2017), and after chronic treatment both decreased the abundance of Firmicutes (Gram-positives) without any significant or reported intestinal injury *in vivo* (Montrose et al., 2016; Lu et al., 2018). By contrast, chronic treatment with rofecoxib, which lacks direct antibacterial effects, did not cause dysbiosis in the uninflamed gut (Lázár et al., 2019).

Another mechanism by which NSAIDs might change the microbiota is the disturbed GI motility. Several studies have demonstrated that NSAIDs can alter intestinal contractility and/or transit time, although there is still no consensus whether this effect is stimulatory or inhibitory. Indomethacin, for example, was shown to both increase (Takeuchi et al., 2002; Nylander, 2011) and decrease small intestinal contractility in the rat (Lichtenberger et al., 2015). Nevertheless, because GI transit time and gut microbiota are highly interrelated, both accelerated and delayed transit may result in dysbiosis (Kashyap et al., 2013). Of note, slow GI transit in loperamide-treated mice was associated with decreased abundance of Firmicutes (e.g. *Lachnospiraceae*) and expansion of Bacteroidetes (Kashyap et al., 2013; Touw et al., 2017), which were also commonly reported following NSAID treatment (Craven et al., 2012; Blackler et al., 2015; Colucci et al., 2018).

Ketorolac, a COX-1-preferential inhibitor (Warner et al., 1999), is mainly used for the short-term treatment of moderate to severe pain in postoperative or emergency patients (Vadivelu et al., 2015). When given at low doses, ketorolac was shown to spare the rat gastric and intestinal mucosa (Jamali et al., 1999; Wallace et al., 2000), despite almost complete suppression of gastric prostaglandin synthesis (Wallace et al., 2000). In addition, in a recent study (Maier et al., 2018) ketorolac did not influence the growth of 40 representative gut bacterial strains *in vitro*.

These properties prompted us to use ketorolac as a pharmacological tool to investigate the relationship between mucosal inflammation and dysbiosis. More specifically, we aimed to determine whether chronic treatment with ketorolac, an NSAID lacking direct effect on gut bacteria, has any significant impact on intestinal microbiota in the absence of mucosal inflammation. Because intestinal bacteria and bile acids are closely interrelated (Begley et al., 2005), we also assessed the effect of ketorolac on small intestinal bile acid composition. In addition, we addressed the possibility that ketorolac-induced bacterial and bile acid alterations are due to a delay in small intestinal transit (“stasis”).

Here we show that ketorolac given at low, non-damaging dose for 4 weeks caused small intestinal dysbiosis, which resembled that caused by other NSAIDs. Namely, it decreased the abundances of bacterial families belonging to Firmicutes, and increased that of *Enterobacteriaceae*. These changes were accompanied by and correlated with significant bile acid alterations. GI transit was not influenced significantly by ketorolac. These results suggest that besides direct effects on bacteria, intestinal inflammation and stasis also other, yet unidentied, factors may contribute to NSAID-induced dysbiosis and bile dysmetabolism.

## Materials and Methods

### Animals

Experiments were carried out on male Wistar rats weighing 180–240 g (Semmelweis University, Budapest, Hungary). Animals were housed in a temperature (22 ± 2°C)- and humidity-controlled room at a 12-h light/dark cycle. Food and water were available *ad libitum* unless otherwise specified.

### Ethical Considerations

All efforts were made to minimize animal suffering and to reduce the number of animals used in the experiments. All procedures conformed to the Directive 2010/63/EU on European Convention for the protection of animals used for scientific purposes. The experiments were approved by the National Scientific Ethical Committee on Animal Experimentation and permitted by the government (Food Chain Safety and Animal Health Directorate of the Government Office for Pest County (PEI/001/1493-4/2015)).

### *In vivo* Studies

#### Study 1. Evaluating the Effects of Chronic Ketorolac Treatment on Small Intestinal Mucosal Integrity, Microbiota and Bile Acids

40 rats were randomly allocated into 4 experimental groups, with 10 rats in each group. In order to control variations in microbiota composition, before the start of the experiment rats were co-housed for 1 week, and during the experiment all groups were divided and housed in 2-2 individually ventillated cages, with 5 rats per cage, to minimize the cage effect (Laukens et al., 2016).

During the experiment, unfasted rats were treated intragastrically with either vehicle (1% hydroxyethylcellulose) or ketorolac tromethamine (1, 3 and 10 mg/kg) (Sigma, St. Louis, MO, United States of America), in a volume of 0.33 ml/100 g. These doses were chosen based on previous studies, showing that ketorolac at doses of ≤3 mg/kg spared the GI mucosa (Jamali et al., 1999; Wallace et al., 2000; Santos et al., 2007), but inhibited gastric prostaglandin synthesis (Wallace et al., 2000). Of note, ketorolac in this dose range has been reported to possess both analgesic and anti-inflammatory properties (Jett et al., 1999). This was also confirmed by our pilot study, in which a 5-day treatment with 1, 3 and 10 mg/kg ketorolac reduced the concentration of PGE_2_ in the carrageenan-airpouch model by 67, 84 and 99%, respectively. Because non-damaging COX-inhibitors (e.g. celecoxib or low-dose ibuprofen) caused intestinal dysbiosis mainly after chronic treatment (1–10 weeks) (Montrose et al., 2016; Lu et al., 2018), we administered ketorolac once daily for 4 weeks. Body weight was measured daily during the course of the treatment. At the end of treatment, 24 h after the final administration of ketorolac, animals were euthanized under CO_2_, the small intestines were excised, the content of distal jejunum was quickly collected, snap-frozen in liquid nitrogen and stored at −80°C for analysis of the microbiota and bile acids. The mucosa of the entire small intestine was flushed with cold saline and examined thoroughly for the presence of macroscopic alterations. The length of the whole small intestine was measured, because bowel shortening correlates with the severity of intestinal inflammation. Because NSAID-induced ulcers in the rat occur predominantly in the middle and distal parts of the small intestine (Kent et al., 1969), full-thickness pieces of the distal jejunum were snap-frozen in liquid nitrogen, pulverized and stored at −80°C for further analysis. A further jejunal segment was fixed in 10% formalin for evaluation of microscopic damage.

#### Study 2. Evaluating the Effect of Ketorolac on GI Transit

Here we aimed to analyze whether ketorolac has any effect on GI peristalsis, which could potentially favor the growth of distinct bacteria and result in dysbiosis after long-term treatment. Rats were treated intragastrically with either vehicle or ketorolac tromethamine (1, 3 and 10 mg/kg) once daily for 5 days, in order to allow any potential drug accumulation or the production of metabolites. On the 5th day, following 18 h fasting and 1 h after the final gavage of vehicle or ketorolac, a charcoal suspension (10% charcoal in 5% gum arabic) was given in a volume of 2 ml/rat per os (Zádor et al., 2020). The timing of charcoal administration was chosen based on pharmacokinetic studies on ketorolac in the rat, showing that maximal blood concentration was achieved within 1 h following per os administration (Granados-Soto et al., 1995; Jamali et al., 1999). 30 min later the rats were euthanized, their entire small intestines were removed and the distance traveled by the charcoal suspension was measured and compared to the total length of small intestine.

### Histological Analysis

Samples taken from the distal part of the small intestine were fixed in 10% formalin, embedded in paraffin, sectioned (5 µm), and stained with hematoxylin and eosin. The severity of epithelial damage, edema and cellular infiltration was analysed by an expert pathologist in a blinded fashion as described before (László et al., 2020). The sections were also digitalized by using a PANNORAMIC Digital Slide Scanner (3DHISTECH Ltd., Budapest, Hungary) in order to take representative images.

### Measurement of Myeloperoxidase and Tumor Necrosis Factor-α by ELISA

ELISA kits were used to quantify the jejunal protein levels of tumor necrosis factor-α (TNF-α) (Invitrogen, Camirillo, CA) and myeloperoxidase (MPO) (Hycult Biotech, Uden, Netherlands). Tissue samples were homogenized and assayed according to the manufacturers’ instructions, in a blinded fashion and in duplicates. The total protein concentration of supernatants was determined by using a bicinchoninic acid assay kit (Thermo Scientific Pierce Protein Research Products, Rockford, United States) with bovine serum albumin as a standard.

### Western Blot Analysis of Cyclooxygenase Isoforms and Claudin-1

Distal jejunal tissues were homogenized with a TissueLyser (Qiagen, Venlo, Netherlands) in lysis buffer containing 200 mM NaCl, 5 mM EDTA, 10 mM Tris, 10% glycerine, and 1 μg/ml leupeptin (pH 7.4), supplemented with a protease inhibitor cocktail (cOmplete ULTRA Tablets, Roche, Basel, Switzerland) and PMSF (Sigma, St. Louis, MO, United States). The homogenized lysates were centrifuged twice at 1500 × g and 4°C for 15 min, then the supernatants were collected and their protein concentration was measured by the bicinchoninic acid assay (Thermo Fisher Scientific, Waltham, MA, United States). Equal amount of protein (20 µg) was mixed with Pierce Lane Marker reducing sample buffer (Thermo Fisher Scientific, Waltham, MA, United States), and loaded and separated in a 4–20% precast Tris-glycine SDS polyacrilamide gel (BioRad, Hercules, CA, United States). Proteins were transferred electrophoretically onto a polyvinylidene difluoride membrane (BioRad, Hercules, CA, United States) at 200 mA overnight. Membranes were blocked with 5% nonfat dry milk (BioRad, Hercules, CA, United States) in Tris-buffered saline containing 0.05% Tween-20 (0.05% TBS-T; Sigma, St. Louis, MO, United States) at room temperature for 2 h. Membranes were incubated with primary antibodies against COX-2 (#12282, 1:500), COX-1 (#4841, 1:500) (Cell Signaling Technology, Danvers, MA, United States) and claudin-1 (ab15098, 1:1000, Abcam, Cambridge, United Kingdom) overnight at 4°C, followed by 2 h incubation at room temperature with anti-rabbit HRP-linked secondary antibody. GAPDH was used to control for sample loading and protein transfer and to normalize the content of target protein. Signals were detected with a chemiluminescence kit (BioRad, Hercules, CA, United States) by Chemidoc XRS+ (BioRad, Hercules, CA, United States).

### DNA Isolation, 16S rRNA Gene Library Preparation and MiSeq Sequencing

Bacterial DNA was extracted from 100 mg small intestinal content per sample using the QIAamp PowerFecal DNA Kit (Qiagen, Hilden, Germany) and further purified using AMPure XP beads (Beckman Coulter, Brea, CA, United States) according to the manufacturer’s protocols. The concentration of genomic DNA was measured using a Qubit 2.0 Fluorometer with Qubit dsDNA HS Assay Kit (Thermo Fisher Scientific, Waltham, MA, United States). Bacterial DNA was amplified with tagged primers (5′-TCG TCG GCA GCG TCA GAT GTG TAT AAG AGA CAG CCT ACG GGN GGC WGC AG and 5′-GTC TCG TGG GCT CGG AGA TGT GTA TAA GAG ACA GGA CTA CHV GGG TAT CTA ATC C), covering the V3-V4 region of the bacterial 16S rRNA gene. PCR and DNA purifications were performed according to Illumina’s demonstrated protocol (Part # 15044223 Rev. B). The PCR product libraries were quantified and qualified by using DNA 1000 Kit on Agilent 2100 Bioanalyzer instrument (Agilent Technologies, Waldbronn, Germany). Equimolar concentrations of libraries were pooled and sequenced on an Illumina MiSeq platform (Illumina, San Diego, CA, United States) using MiSeq Reagent Kit v3 (600 cycles PE).

Essentially the bioinformatic analysis was carried out as described by Mansour et al. (2020). Briefly, the quality of raw reads was assed with FastQC and MultiQC38, the low quality sequences were filtered and trimmed by Trimmomatic (Bolger et al., 2014) and only sequences with minimal length of 36 were kept, and the low quality base calls were discarded (phred score < 20). The SSU Ref NR 99 database (release 132) of SILVA (Quast et al., 2013) was used, which was preprocessed and indexed by the Kraken2 (Wood and Salzberg, 2014) with k-mer = 31. The final microbiome composition was estimated by Bracken (Breitwieser et al., 2019).

### Measurement of Small Intestinal Bile Acids by LC-MS/MS

Luminal samples (100 mg on average) obtained from the distal small intestine were sent to Biocrates Life Science AG (Innsbruck, Austria) on dry ice, where metabolomic profiling was performed. Samples were weighed, mixed with the 3-fold volume of extraction buffer (85% ethanol, 15% 0.01 M phosphate buffer (pH 7.4)), and vortexed thoroughly until dissolution. Samples were then sonicated and centrifuged, and the supernatant was used for further analysis. Intestinal luminal bile acids were assessed by electrospray (ESI) mass spectrometry coupled to liquid chromatography (LC) with a tandem mass spectrometry (MS/MS) instrument (SCIEX 4000 QTRAP^®^,SCIEX, Darmstadt, Germany), by using the commercially available Biocrates® Bile Acids Kit (Biocrates Life Science AG, Innsbruck, Austria), which enabled the absolute quantification of 20 different bile acids (Table 1). A highly selective reversed phase high-performance liquid chromatography-tandem mass spectrometry (LC-MS/MS) analysis method in negative ion multiple reaction monitoring detection mode was applied to determine the concentrations of bile acids The experimental metabolomics measurement technique is described in detail by patent US 2007/0004044 (accessible online at http://www.freepatentsonline.com/20070004044.html). For highly accurate quantification, 7-point external calibration curves and 10 stable isotope-labeled internal standards were applied. Data of bile acids were quantified using the appropriate MS software (SCIEX - Analyst) and the results were finally imported into Biocrates MetIDQ software for further analysis.

**TABLE 1:** The list of measured bile acids.

Abbreviation	Name	Type
CA	Cholic acid	Primary
CDCA	Chenodeoxycholic acid	Primary
DCA	Deoxycholic acid	Secondary
GCA	Glycocholic acid	Primary, glycine-conjugated
GCDCA	Glycochenodeoxycholic acid	Primary, glycine-conjugated
GDCA	Glycodeoxycholic acid	Secondary, glycine-conjugated
GUDCA	Glycoursodeoxycholic acid	Secondary, glycine-conjugated
HDCA	Hyodeoxycholic acid	Secondary
LCA	Lithocholic acid	Secondary
MCA(α)	α-Muricholic acid	Primary
MCA(β)	β-Muricholic acid	Primary
MCA(ω)	ω-Muricholic acid	Secondary
TCA	Taurocholic acid	Primary, taurine-conjugated
TCDCA	Taurochenodeoxycholic acid	Primary, taurine-conjugated
TDCA	Taurodeoxycholic acid	Secondary, taurine-conjugated
TLCA	Taurolithocholic acid	Secondary, taurine-conjugated
TMCA (α + β)	α- and β-Tauromuricholic acid	Primary, taurine-conjugated
TUDCA	Tauroursodeoxycholic acid	Secondary, taurine-conjugated
UDCA	Ursodeoxycholic acid	Secondary

The mean hydrophobicity index of the luminal content was calculated as a percentage-weighted mean of previously reported hydrophobicities of the individual bile acids (Heuman, 1989; Stenman et al., 2013; Poša, 2014).

### Data Analysis

Statistical analysis of the data was performed with Student t test or Mann-Whitney U test (in case of nonparametric values), or with one-way ANOVA, followed by Holm-Sidak post hoc test. Two-way repeated measures ANOVA was employed to compare the time course of weight losses. Outliers detected by Grubb’s test were excluded from the analyses. A probability of *p* < 0.05 was considered statistically significant.

Alpha diversities were quantified by using the Shannon index (quantifying entropy of the distribution of taxa proportions). Taxa having at least support of 50 reads were considered as positive, others were discarded from the downstream analysis. Principal component analysis (PCA) was used for visualizing the microbiome composition. The differential abundance tests were performed using ANCOM (Mandal et al., 2015) with Holm-Bonferroni corrected alpha of 0.05 as the rejection threshold. Zero abundance of taxa was replaced by a pseudocount 1.

Associations between bacterial abundances and absolute and relative bile acid concentrations were tested by calculating Spearman’s rank correlation coefficients (Rho-value) for each pair of these variables across all samples. Correlation test *p*-values were corrected for type I error by applying the false discovery rate method (FDR; q-value according to Benjamini and Hochberg (1995)), and significant correlations were declared in case of q < 0.05. Heat maps with rows and columns ordered by Euclidian distance-based complete linkage clustering and corresponding dendrograms were generated to visualize Rho-values of the pairwise correlation tests. Zero row and column vectors were dropped from the heatmaps. All steps of the correlation analysis were performed with the use of the R software package (version 3.4.4) (R Core Team, 2018).

## Results

### Ketorolac up to 3 mg/kg did not Cause Significant Damage to the Small Intestinal Mucosa

**FIGURE 1:**
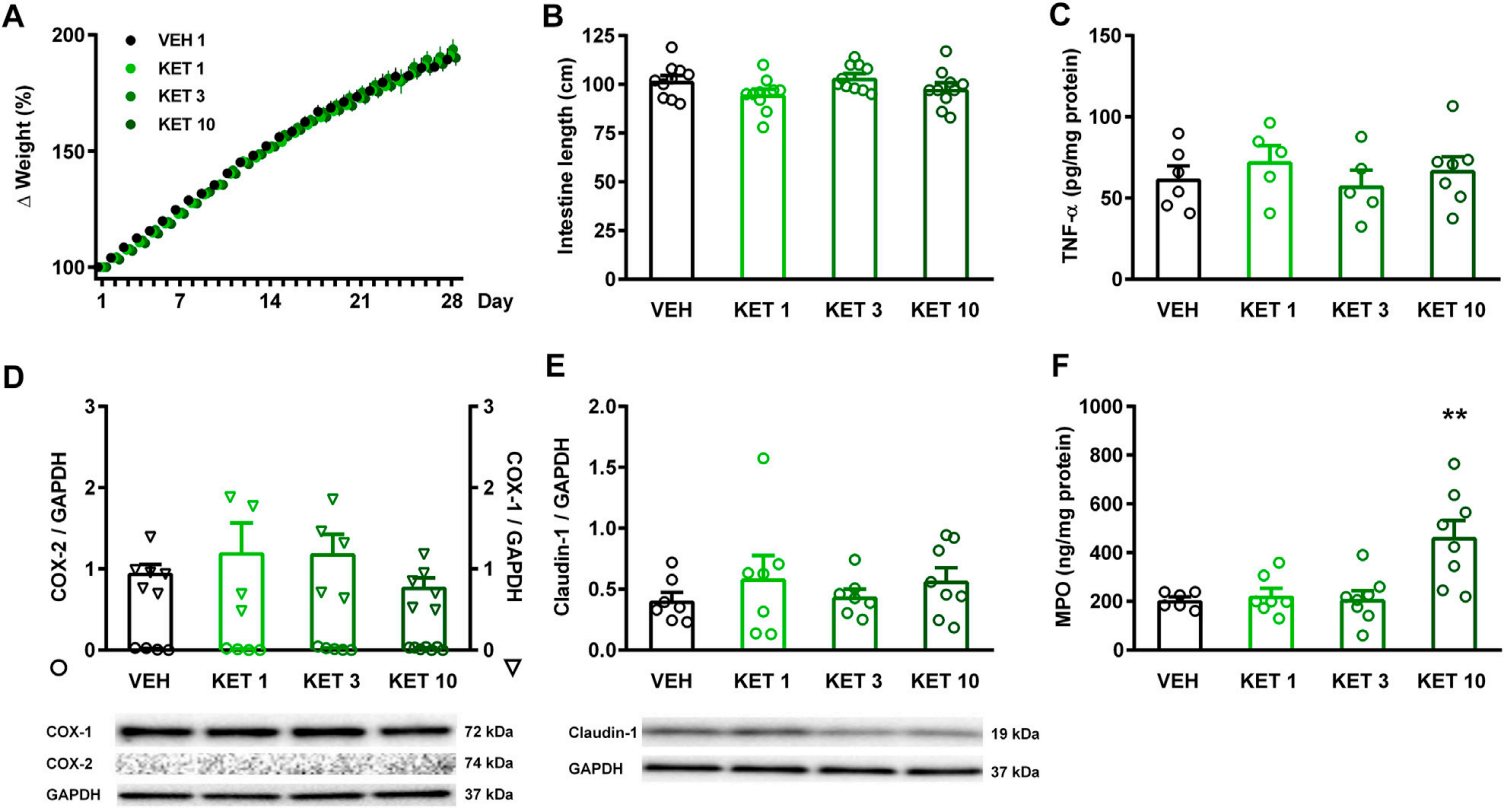
The effect of ketorolac (KET) given at different doses (1, 3 and 10 mg/kg per os, once daily for four weeks) on animal weight, small intestine length, and expression of inflammatory mediators and claudin-1. Panel **A**: results are expressed as the mean + SEM. Panels **B–F**: Circles represent the data of each rat, bars indicate the mean + SEM. For statistical analysis two-way repeated measures ANOVA followed by Holm-Sidak post hoc test **(A)**, and one-way ANOVA with Holm-Sidak post hoc test **(B–F)** were used, n = 4–10/group. **p < 0.01 compared to vehicle-treated group.

None of the rats died over the 4-week treatment period, and none of the tested doses of ketorolac caused weight loss compared to vehicle (Figure 1A). Upon sacrificing the animals, the mucosa of the entire small intestine was examined thoroughly, but there were no visible morphologic alterations, such as bleeding or ulcers. There were no differences in terms of intestinal lengths, indicating that ketorolac treatment did not cause bowel shortening either (Figure 1B). We have also performed a gross examination of the gastric mucosa, but it did not reveal any significant damage (not shown).

The presence of inflammation was assessed by measuring the tissue protein levels of TNF-α, COX-2 and MPO (Figures 1C,D,F). COX-2 expression was not detectable by Western blotting in any of the samples, in contrast to the abundant expression of the constitutive COX-1 isoform. Likewise, ketorolac treatment had no effect on the expression of TNF-α, measured by ELISA. The level of MPO, however, was significantly increased in the small intestine of rats treated with the highest dose of ketorolac. Because a decrease in key tight junction proteins, including claudin-1, corresponds to an increase in intestinal permeability (Han et al., 2016; Xiao et al., 2017), we also measured the expression of claudin-1 to assess whether ketorolac treatment has any effect on intestinal barrier. As Figure 1E shows, we did not found difference between the claudin-1 expression of vehicle- and ketorolac-treated animals.

**FIGURE 2:**
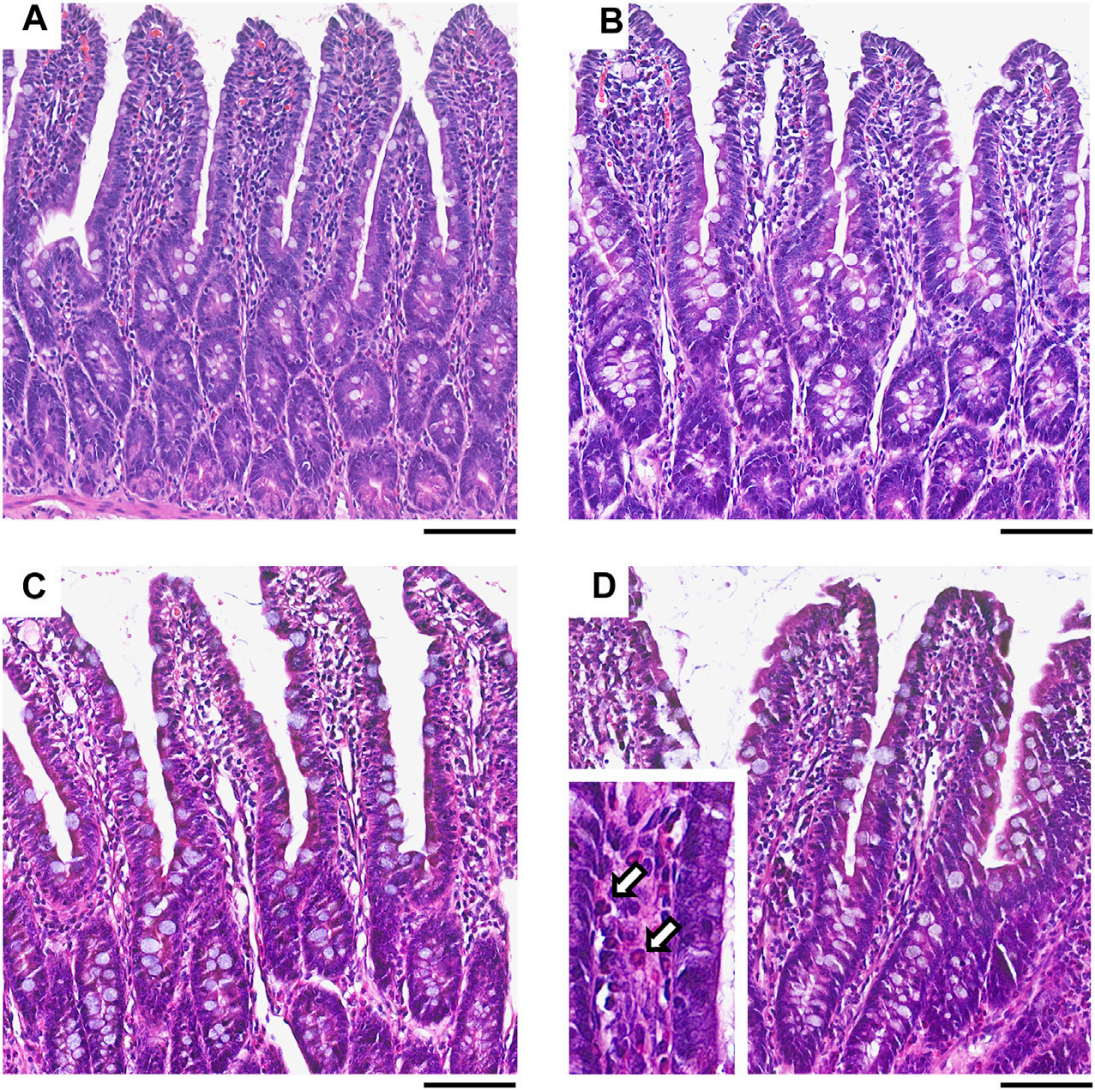
Representative histological micrographs of the distal jejunum of rats treated with vehicle **(A)**, or ketorolac at doses of 1 **(B)**, 3 **(C)** and 10 mg/kg **(D)** for four weeks (scale bar: 100 μM, hematoxylin and eosin staining). White arrows point to neutrophil granulocytes, indicating increased cellularity of the lamina propria.

Collectively, these data indicate that ketorolac at doses of 1 and 3 mg/kg did not cause significant damage to the small intestine, whereas at a dose of 10 mg/kg ketorolac caused mild inflammation with elevation of MPO. This was also confirmed by histological analysis of the intestinal samples. The structure of villi and epithelium were generally preserved in all rats. Granulocytes were sparsely present in the lamina propria of most samples, but the highest number of cells was noted in the 10 mg/kg group (Figure 2).

### Ketorolac at a Dose of 3 mg/kg Induced Small Intestinal Dysbiosis

**FIGURE 3:**
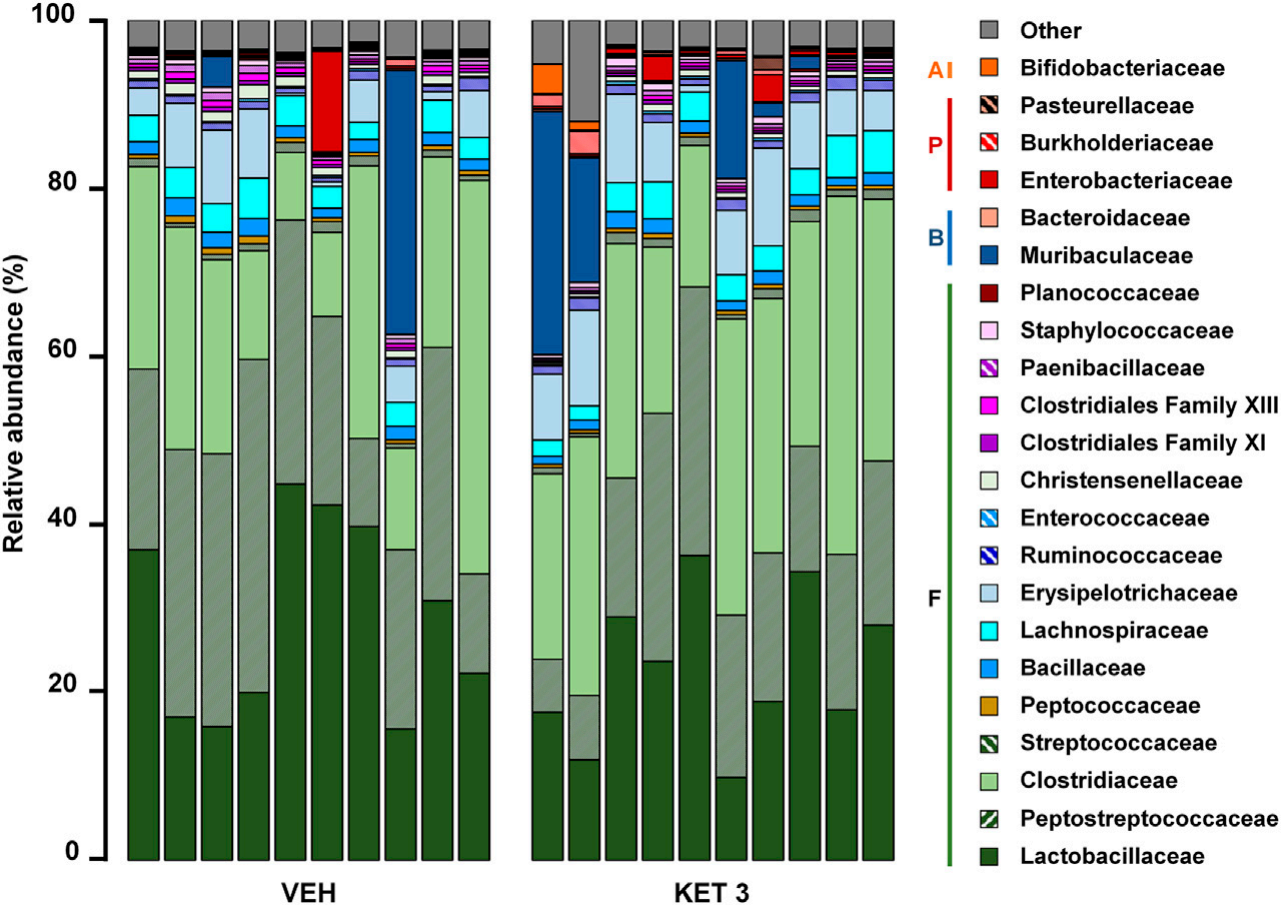
The relative abundance of bacterial families in jejunal samples of rats treated with vehicle (VEH) or ketorolac (KET, 3 mg/kg) for four weeks, determined by sequencing of 16 S rRNA. Each vertical bar represents the sequencing data for one rat. Unclassified families and families with an abundance less than 0.1% are summarized as “Other.” Abbreviations: F: Firmicutes, B: Bacteroidetes, P: Proteobacteria, A: Actinobacteria.

**FIGURE 4:**
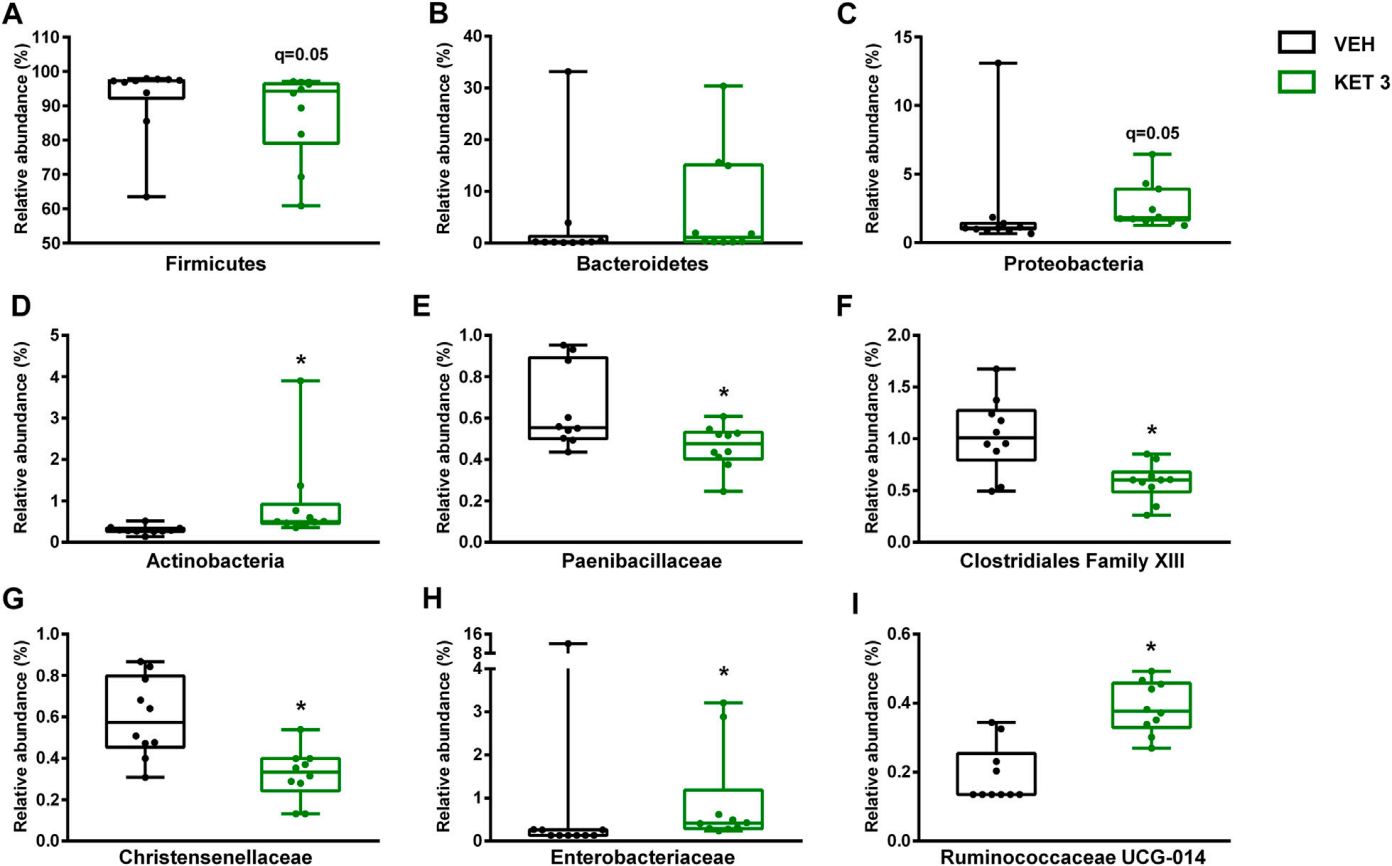
The effect of four-week vehicle (VEH) or ketorolac treatment (KET, 3 mg/kg) on the small intestinal proportion of different bacterial phyla **(A–D)**, families **(E–H)** and the genus *Ruminococcaceae UCG-014* **(I)**. Box and whisker plots indicate the medians, first and third quartiles, and the minimum and maximum values, the points represent individual samples. The differential abundance tests were performed using ANCOM with Holm-Bonferroni correction for multiple comparison, n = 10/group. *q < 0.05 compared to vehicle-treated group.

We next examined whether ketorolac at the dose of 3 mg/kg (the highest tested non-damaging dose) has any effect on the composition of small intestinal microbiota. In control rats, the vast majority of taxa belonged to Firmicutes (92.5%), followed by Bacteroidetes (3.9%), Proteobacteria (2.3%) and Actinobacteria (0.3%). At the family level, the most abundant bacterial families were *Lactobacillaceae*, *Peptostreptococcaceae* and *Clostridiaceae* (Figure 3). Although the same taxa dominated the microbiota of ketorolac-treated rats (Figure 3), there were notable differences in their proportions compared to the vehicle group. As Figure 4 shows, at the phylum level ketorolac increased the abundance of Actinobacteria, and tended to increase that of Bacteroidetes and Proteobacteria. By contrast, the level of Firmicutes tended to decrease, which was mainly due to the decreased level of the families *Paenibacillaceae*, *Clostridiales Family XIII* and *Christensenellaceae*. In addition, ketorolac treatment was associated with higher levels of *Enterobacteriaceae*. Among the identified genera we found significant difference only between the levels of *Ruminococcaceae UCG-014* (Figure 4), which was increased in the ketorolac group.

**FIGURE 5:**
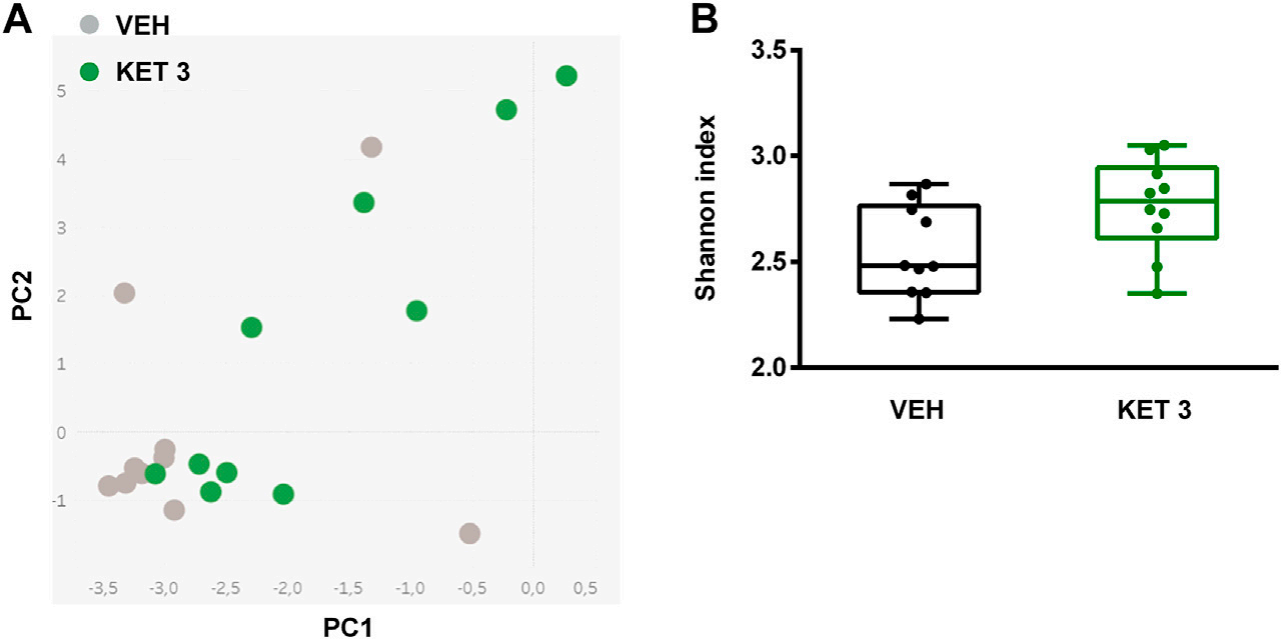
Panel **A**: Principal component analysis (PCA) score plot comparing the small intestinal microbiota composition of rats treated with either vehicle (VEH) or ketorolac (KET, 3 mg/kg) for four weeks. Panel **B**: The effect of ketorolac on small intestinal bacterial diversity, estimated by the Shannon index. Box and whisker plots indicate the medians, first and third quartiles, and the minimum and maximum values, the points represent individual samples. For statistical analysis Mann-Whitney U test was used, n = 10/group.

Hence, despite the lack of mucosal injury ketorolac induced significant changes in the composition of small intestinal microbiota. This was also confirmed by principal component analysis (PCA) of the data, in which a clear shift of ketorolac samples was observed on both axes (Figure 5A). Bacterial diversities were estimated by calculating the Shannon indices, which were not different between the two groups (Figure 5B).

### Ketorolac at a Dose of 3 mg/kg Altered the Small Intestinal Bile Acid Composition

**FIGURE 6:**
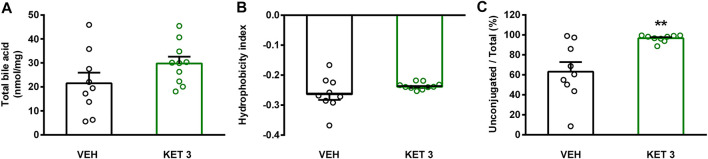
The effect of four-week vehicle (VEH) or ketorolac treatment (KET, 3 mg/kg) on small intestinal total ileal bile acid level **(A)**, mean hydrophobicity index **(B)**, and ratio of conjugated to total ileal bile acids **(C)**. Circles represent the data of each rat, bars indicate the mean + SEM. For statistical analysis Student’s t test was used, n = 9–10/group. **p < 0.01 compared to vehicle-treated group.

**FIGURE 7:**
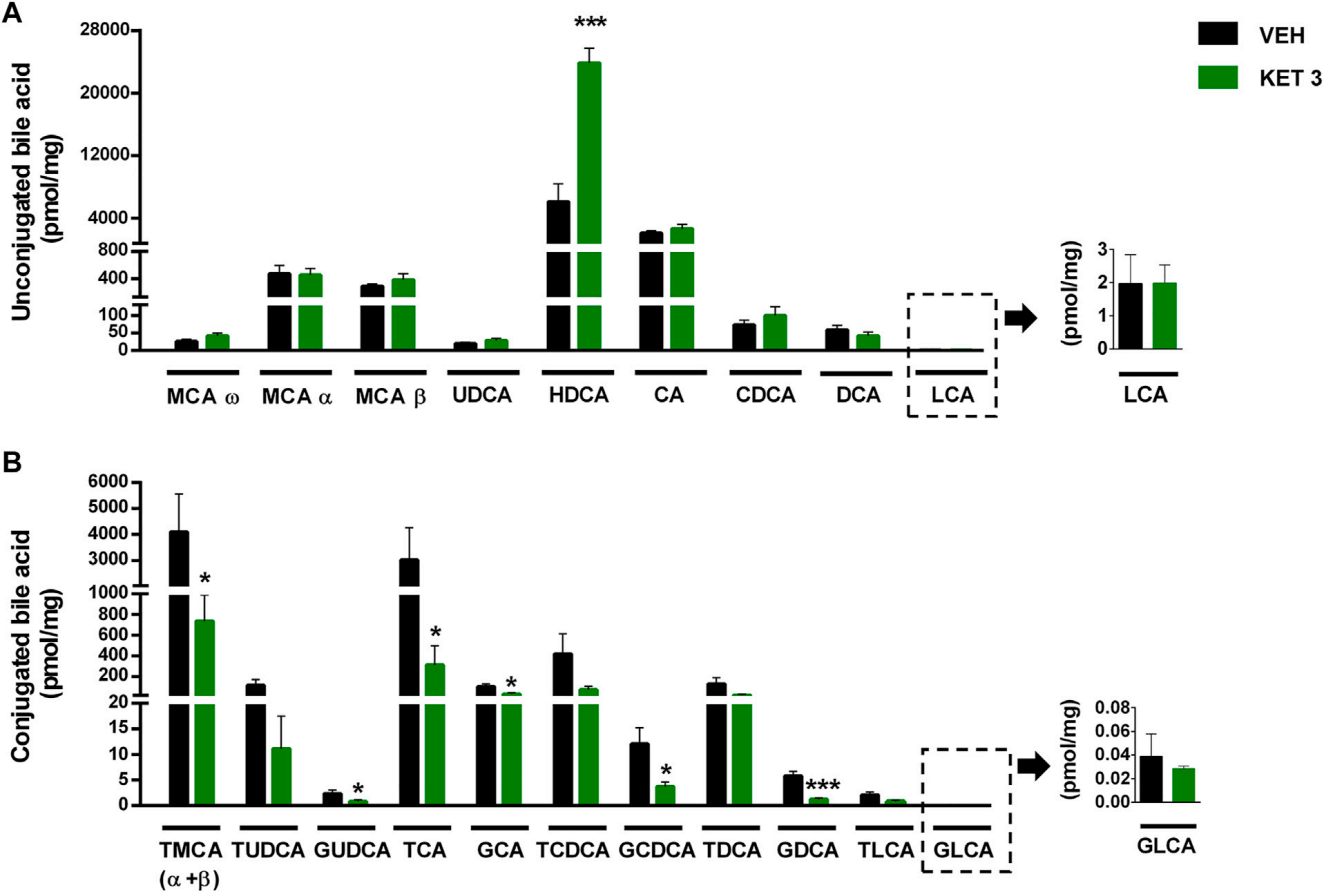
The effect of four-week vehicle (VEH) or ketorolac treatment (KET, 3 mg/kg) on the small intestinal concentration of **(A)** unconjugated and **(B)** conjugated bile acids. Bile acids are ordered by increasing hydrophobicity. Bars indicate the mean + SEM, for statistical analysis Student’s t test was used, n = 9–10/group. *p < 0.05, ***p < 0.001 compared to vehicle-treated group. MCA, muricholic acid; UDCA, ursodeoxycholic acid; HDCA, hyodeoxycholic acid; CA, cholic acid; CDCA, chenodeoxycholic acid; DCA, deoxycholic acid; LCA, lithocholic acid; T, respective taurine conjugates; G, respective glycine conjugates.

The luminal concentration of bile acids in the distal small intestine was measured by LC-MS/MS (Supplementary Table S1). As Figure 6 shows, ketorolac treatment changed neither the total concentration of bile acids, nor the overall hydrophobicity of small intestinal bile. By contrast, it increased dramatically the ratio of unconjugated to total bile acids. This was due to an increase in the concentration of the secondary bile acid hyodeoxycholic acid (HDCA), and a decrease in the levels of glycine- and taurine-conjugated primary and secondary bile acids (Figure 7). We found no differences between the concentrations of any other unconjugated primary and secondary bile acids in the two groups.

### Ketorolac-Induced Dysbiosis Correlated with the Changes of Bile Acids

**FIGURE 8:**
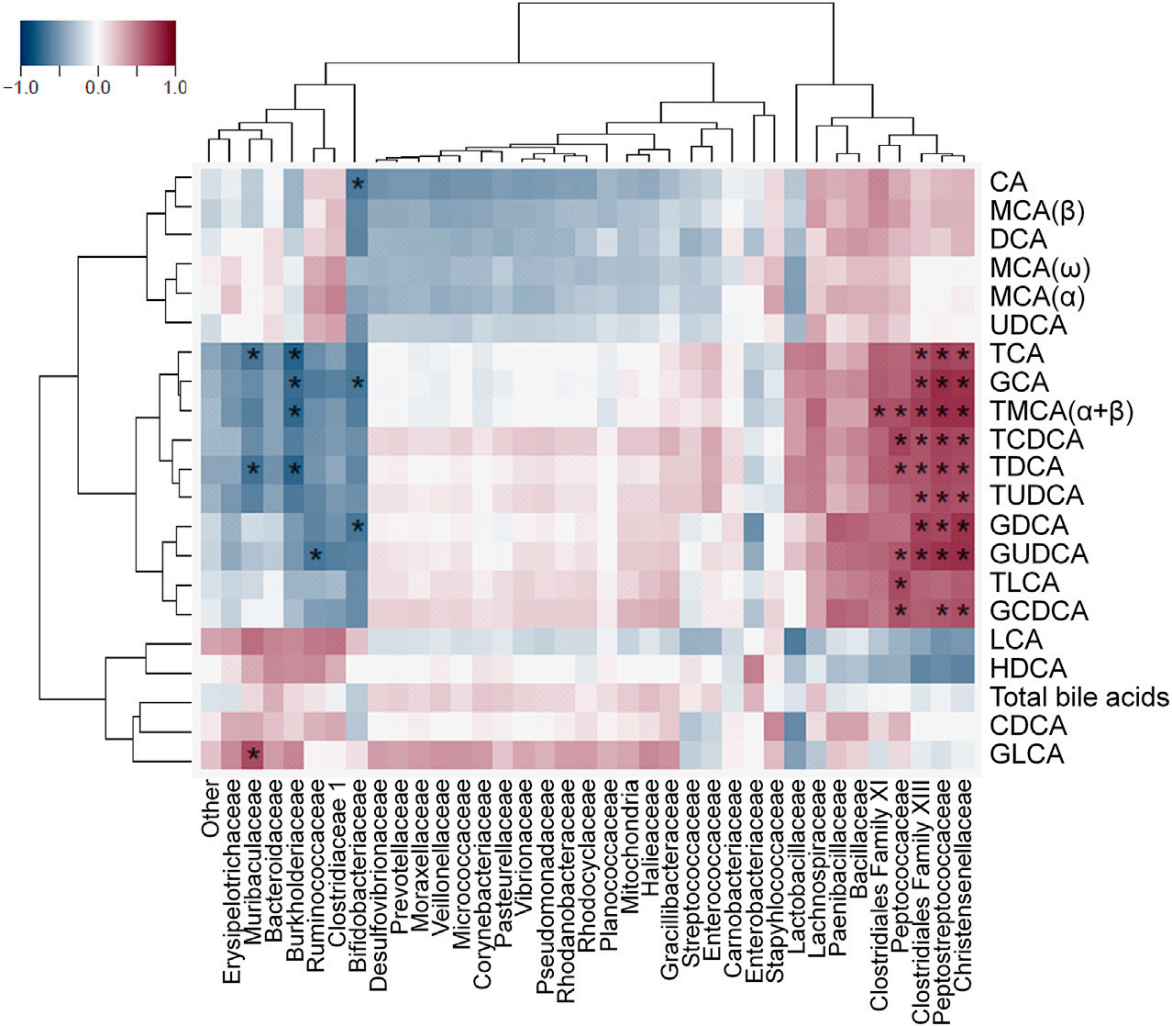
Heat map of Spearman’s correlation coefficients between the small intestinal concentration of individual bile acids and the relative abundance of bacterial families. Individual *p* values were corrected by the false discovery rate method according to Benjamini and Hochberg, asterisks indicate q < 0.05.

Spearman's correlation analysis was performed to explore the associations between ketorolac-induced dysbiosis and bile dysmetabolism, and calculated Rho-values were visualized as heat maps with dendrograms to identify related variables. Some bacterial families, such as *Erysipelotrichaceae*, *Ruminococcaceae*, *Clostridiaceae 1* (all belonging to Firmicutes), *Muribaculaceae*, *Bacteroidaceae* (both belonging to Bacteroidetes), *Burkholderiaceae* and *Bifidobacteriaceae* clustered together and correlated negatively with the absolute concentrations and proportions of glycine- and taurine conjugates (Figure 8 and Supplementary Figure S1). The family *Burkholderiaceae* also showed positive correlation with the percentage of HDCA. By contrast, other Firmicutes bacteria (*Clostridiales Family XI* and *XIII*, *Peptocococcaceae*, *Peptostreptococcaceae*, *Christensenellaceae*) formed another cluster and showed positive correlation with conjugated bile acids, and negative with HDCA (Figure 8 and Supplementary Figure S1).

### Ketorolac had no Significant Effect on GI Transit

**FIGURE 9:**
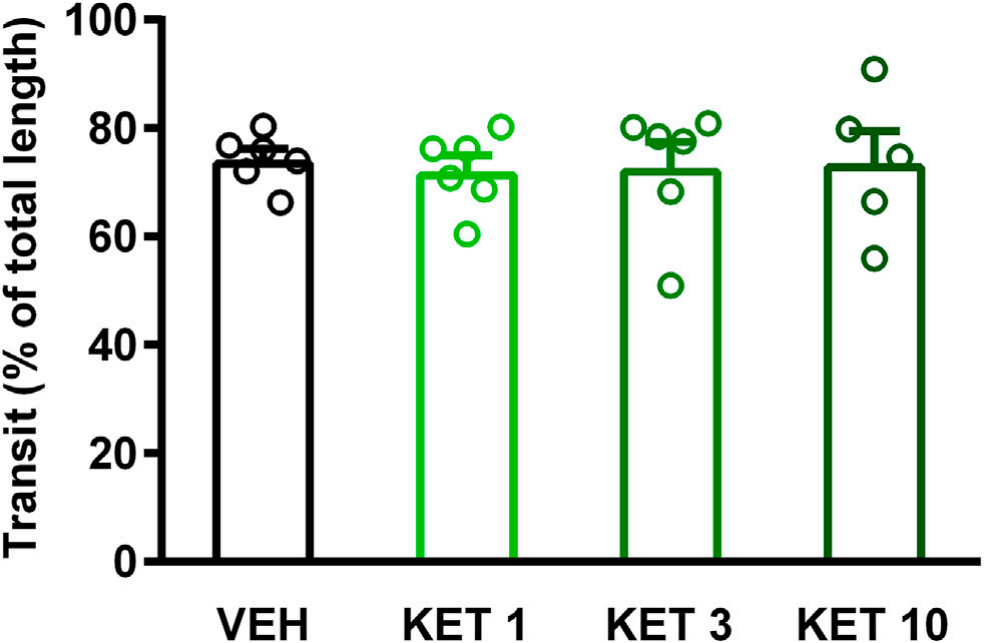
The effect of vehicle (VEH) or ketorolac (KET) given at different doses (1, 3 and 10 mg/kg per os, once daily for five days) on rat gastrointestinal transit. Circles represent the distance traveled by charcoal in each rat, bars indicate the mean + SEM. For statistical analysis one-way ANOVA with Holm-Sidak post hoc test was used, n = 5–6/group.

Small intestinal stasis has long been known to favor the growth of *Bacteroides* and deconjugation of bile acids (Gorbach and Tabaqchali, 1969). Therefore, we aimed to investigate whether delayed intestinal peristalsis contributes to the observed changes of bacteria and bile acids. As Figure 9 shows, ketorolac treatment did not change the GI transit of the charcoal suspension.

## Discussion

Here we report that small intestinal dysbiosis developed despite any significant mucosal injury and inflammation in rats treated with low-dose ketorolac, an NSAID lacking direct effects on gut bacteria. The observed bacterial changes were similar to those reported in previous studies on NSAID enteropathy, characterized by a loss of families belonging to Firmicutes and bloom of *Enterobacteriaceae*. Our results indicate that other factors than mucosal inflammation may contribute to NSAID-induced dysbiosis as well. The role of altered intestinal peristalsis is unlikely, because ketorolac had no significant impact on GI transit. In addition, ketorolac-induced dysbiosis was accompanied by and correlated with significant changes in the intestinal composition of bile acids, which were characterized by reduction of conjugated bile acids and elevation of HDCA.

Although the influence of NSAIDs on the composition of intestinal microbiota is known since several decades (Kent et al., 1969), the underlying mechanism is still poorly understood. Intestinal injury caused by NSAIDs is undoubtedly a major factor driving the changes of microbiota, because the severity of enteropathy correlates with the extent of dysbiosis (Reuter et al., 1997; Craven et al., 2012). Moreover, the inflamed microenvironment promotes the growth of *Enterobacteriaceae* (Zeng et al., 2017), and the expansion of this bacterial family is common following NSAID treatment (Kent et al., 1969; Craven et al., 2012; Terán-Ventura et al., 2014; Blackler et al., 2015). Another factor that may potentially contribute to the NSAID-induced shift in the microbiota toward Gram-negative bacteria is an antibacterial activity against Gram-positives, which has been reported *in vitro* for several NSAIDs, including ibuprofen, diclofenac and celecoxib (Obad et al., 2015; Thangamani et al., 2015; Chan et al., 2017). Of note, ketorolac, a COX-1 preferring NSAID (Warner et al., 1999), had no significant effect on the growth of 40 representative gut bacterial strains *in vitro* (Maier et al., 2018). Our results that chronic treatment with ketorolac at non-damaging dose altered the intestinal microbiota provides evidence for the contribution of additional factors to NSAID-induced dysbiosis.

Although the non-damaging dose of ketorolac (3 mg/kg) used for analysis of bacteria and bile acids was chosen based on previous publications (Jamali et al., 1999; Wallace et al., 2000; Santos et al., 2007), the first and most critical step was to confirm its GI safety. The effect of ketorolac given at different doses on the small intestine was evaluated by macroscopic examination of the entire intestinal mucosa, histological analysis and measurement of inflammatory mediators and claudin-1. Our results that ketorolac at doses up to 3 mg/kg did not cause significant GI damage in the rat correspond to literature data (Jamali et al., 1999; Wallace et al., 2000; Santos et al., 2007). Of note, in cats the same dose of ketorolac induced ulcers in the duodenum, although not in other parts of the small intestine (Satoh et al., 2013), suggesting species differences in its GI tolerability.

Despite no signs of injury we found overt changes in the small intestinal microbiota following ketorolac treatment, which was primarily characterized by a loss of Firmicutes and increased representation of *Enterobacteriaceae*. This result corroborates some previous findings that NSAID-induced dysbiosis can appear without the development of enteropathy as well (Uejima et al., 1996; Liang et al., 2015; Montrose et al., 2016; Lu et al., 2018). These studies, however, focused mainly on the NSAID-induced compositional changes of microbiota, and not on the relationship between inflammation and dysbiosis, therefore only morphological alterations of the intestinal mucosa were assessed. In addition, such NSAIDs were used that may potentially influence the growth of bacteria directly. Our current analysis using ketorolac clearly indicates that NSAID-induced dysbiosis can develop without mucosal inflammation, and based on the *in vitro* results of Maier et al. (2018) also the role of direct effects on gut bacteria is unlikely.

In some (Bennett et al., 1976; Shahbazian et al., 2001; Lichtenberger et al., 2015), but not in other (Takeuchi et al., 2002; Satoh et al., 2009) studies NSAIDs were shown to suppress intestinal motility. Because slower peristalsis was associated with a decrease in Firmicutes and an increase in Bacteroidetes (Kashyap et al., 2013; Touw et al., 2017), we hypothesized that ketorolac induced bacterial changes may be due at least partly to delayed GI peristalsis. However, we found no significant change in GI transit following ketorolac treatment. Of note, in the study of Santos et al. (2007), ketorolac delayed the peristalsis of proximal and medial small intestine. Nevertheless, this effect was detectable only 10 and 20, but not 30, minutes after the application of the test meal. Here we evaluated the transit of test meal 30 min after the gavage, in an attempt to reveal perturbations in peristalsis in more distal parts of the small intestine, at the site of dysbiosis. The lack of effect at this time point (in both studies) indicates that the effect of ketorolac on small intestinal motility at low doses is moderate at best, and does not affect the transit in distal parts of the small intestine. Hence, it is unlikely that altered peristalsis would account for the observed shift in the distal small intestinal microbiota following ketorolac treatment. Similar conclusion was drawn by Hagiwara et al. (2004), who found that DuP-697, a selective COX-2 inhibitor, induced dysbiosis without suppression of peristalsis. A potential limitation of our study is that small intestinal peristalsis was measured only by the charcoal method. Although this simple technique is widely used to assess the net effect of drugs on small intestinal transit, it does not allow to determine the contractile and peristaltic activities of different intestinal segments. Hence, we cannot exclude the possibility that ketorolac had diverse effects on the contractility of proximal, medial and distal parts of the small intestine, resulting in an apparently unchanged transit at the time of evaluation. Further studies are needed to reveal such effects and their potential contribution to small intestinal dysbiosis.

There is a large body of evidence on the close bidirectional relationship between gut bacteria and bile acids. The gut microbiota is capable of producing a wide variety of bile acid metabolites, which may also have diverse effects on the growth of different bacteria (Begley et al., 2005; Ridlon and Bajaj, 2015; Ridlon et al., 2016). Hence, we also aimed to assess whether ketorolac-induced dysbiosis and alterations in the availability of bacterial enzymes had any effect on intestinal bile composition. We found that ketorolac decreased the amount of glycine- and taurine-conjugated primary and secondary bile acids, and increased the concentration of the hydrophilic secondary bile acid HDCA. To date, there is only limited data on the effect of NSAIDs on bile acid composition. Because indomethacin was shown to increase the biliary proportion of hydrophobic bile acids and bile hydrophobicity (Yamada et al., 1996; Uchida et al., 1997), and because the hydrophobicity of bile acids correlates with their cytotoxicity (Heuman, 1989), it was postulated that NSAID-induced small intestinal damage may be partly due to an increased ratio of hydrophobic secondary bile acids (Yamada et al., 1996; Wallace, 2013; Montenegro et al., 2014; Syer et al., 2015). However, in a recent paper focusing mainly on the combined toxicity of ibuprofen and glucocorticoids (Lu et al., 2018), ibuprofen treatment had no effect on the total amount secondary bile acids in the ileum of mice, but increased the amount of primary and conjugated bile acids. Moreover, our recent studies with high-dose indomethacin showed that in severe enteropathy the levels of all conjugated bile acids increased irrespective of their primary or secondary nature, whereas the levels of unconjugated bile acids decreased, except that of HDCA (Lázár et al., submitted manuscript)^1^. Hence, NSAIDs may have diverse effects on small intestinal bile composition, depending on the presence and severity of mucosal damage and the actual microbiota, and the elevation of hydrophobic secondary bile acids is probably not a general consequence of NSAID treatment. On the other hand, NSAIDs can also increase the cytotoxicity of bile due to the formation of more toxic mixed micelles (Barrios and Lichtenberger, 2000; Petruzzelli et al., 2006; Petruzzelli et al., 2007; Zhou et al., 2010; Dial et al., 2015). Therefore, we cannot rule out the possibility that ketorolac treatment increased the toxicity of bile despite having no effect on the overall hydrophobicity of bile acids, but even if this is the case, it still did not cause significant mucosal damage.

The reduced level of conjugated bile acids was likely due to the overgrowth of bacteria involved in bile acid deconjugation. Indeed, deconjugation of bile acids is mainly performed by members of Firmicutes (e.g. *Clostridium*, *Ruminococcus*), Bacteroidetes and *Bifidobacteriaceae* (belonging to Actinobacteria) (Ridlon et al., 2016; Martin et al., 2018), and ketorolac treatment was associated with increased abundance of Actinobacteria and *Ruminococcaceae UCG-014*, and tendency toward elevation of Bacteroidetes. In addition, our correlation analysis revealed negative associations between the levels of conjugated bile acids and the abundance of some Firmicutes (*Erysipelotrichaceae*, *Ruminococcaceae*, *Clostridiaceae 1*), *Muribaculaceae* and *Bacteroidaceae* (both belonging to Bacteroidetes) and *Bifidobacteriaceae*. Although we also found positive correlations between other Firmicutes bacteria (*Clostridiales Family XI* and *XIII*, *Peptocococcaceae*, *Peptostreptococcaceae*, *Christensenellaceae*) and conjugated bile acids, these reflect most likely the loss of these bacteria due to ketorolac treatment in parallel with the reduction of conjugated bile acids and lack causality.

At present, little is known about the bacteria involved in the formation of HDCA. Eyssen et al. (1999) reported the isolation of a Gram-positive rod (termed *HDCA-1*) capable to produce HDCA in the presence of an unidentified growth factor produced by a *Ruminococcus productus* strain. Trudeau et al. (2018) recently found positive correlation between the levels of HDCA and *Lachnospiraceae* in pigs. In the present study, the percentage of HDCA showed the strongest positive correlation with the abundance of *Burkholderiaceae*. Whether this association is due to a causal relationship warrants further investigation.

Although the mild changes in small intestinal microbiota and bile acids due to chronic ketorolac treatment were not translated into enteropathy, they may have other important effects on the host. For example, a decreased ratio of Firmicutes to Bacteroidetes may decrease the capacity to harvest energy from the diet (Turnbaugh et al., 2006), and also the increased luminal concentration of HDCA may have glucose-lowering and obesity-preventing effects (Shih et al., 2013). Nevertheless, these potential favourable effects have probably limited, if any, translational relevance due to the GI damaging property and short-term use of ketorolac in humans.

In conclusion, our present study demonstrates that ketorolac, an NSAID lacking direct antibacterial properties, given at low dose caused marked changes in the composition of small intestinal bacteria in rats without inducing significant mucosal inflammation and tissue damage. This dysbiosis was accompanied by and correlated with significant changes in the intestinal composition of bile acids. Our result, that ketorolac had no significant effect on GI peristalsis, suggests that dysbiosis is not likely to be caused by small intestinal stasis, and other, yet unidentified, factors contribute to the observed changes of bacteria and bile acids. Further studies are warranted to reveal the exact mechanisms underlying the associations between NSAIDs, bacteria and bile acids.

## Data Availability

The datasets presented in this study can be found in online repositories. The names of the repository/repositories and accession number(s) can be found below: Bioproject: https://www.ncbi.nlm.nih.gov/bioproject/PRJNA705956, Zenodo: https://zenodo.org/record/4601191.

## Footnotes

1 Lázár, B., Brenner, G., Makkos, A., Balogh, M., László, S., Al-Khrasani, M., et al. (2019). Lack of Small Intestinal Dysbiosis Following Long-Term Selective Inhibition of Cyclooxygenase-2 by Rofecoxib in the Rat. Cells 8 (3), 251. doi:10.3390/cells8030251
