# Supplementary material for: The Nonsteroidal Anti-Inflammatory Drug Ketorolac Alters the Small Intestinal Microbiota and Bile Acids Without Inducing Intestinal Damage or Delaying Peristalsis in the Rat
Source: Front Pharmacol. 2021 Jun 4;12:664177. doi: 10.3389/fphar.2021.664177 (PMC8213092; doi:10.3389/fphar.2021.664177)
Supplement: Supplementary file 1 [file Table1.pdf]

frontiers

## *Supplementary Table 1*

# The nonsteroidal anti-inflammatory drug ketorolac alters the small intestinal microbiota and bile acids without inducing intestinal damage or delaying peristalsis in the rat

Barbara Hutka, Bernadette Lázár, András S. Tóth, Bence Ágg, Szilvia B. László, Nóra Makra, Balázs Ligeti, Bálint Scheich, Kornél Király, Mahmoud Al-Khrasani, Dóra Szabó, Péter Ferdinandy, Klára Gyires, Zoltán S. Zádori*

*** Correspondence:**
Dr. Zoltán S. Zádori
zadori.zoltan@med.semmelweis-univ.hu

**Table S1. The luminal concentration of measured bile acids in the small intestine of vehicle- (VEH) and ketorolac-treated (KET, 3 mg/kg) rats.** Values are expressed in pmol/mg weighed portion. MCA, muricholic acid; UDCA, ursodeoxycholic acid; HDCA, hyodeoxycholic acid; CA, cholic acid; CDCA, chenodeoxycholic acid; DCA, deoxycholic acid; LCA, lithocholic acid; T, respective taurine conjugates; G, respective glycine conjugates.

| Sample No | Treat-ment | CA | CDCA | DCA | GCA | GCDCA | GDCA | GLCA | GUDCA | HDCA | LCA |
|---|---|---|---|---|---|---|---|---|---|---|---|
| 1 | VEH | 2373 | 56,2 | 40,9 | 83,5 | 4,682 | 4,040 | 0 | 1,3504 | 157,0 | 0,2192 |
| 2 | VEH | 5467 | 141,6 | 98,2 | 78,0 | 5,600 | 4,376 | 0,0244 | 1,6976 | 310,8 | 0,5132 |
| 3 | VEH | 3053 | 118,6 | 61,4 | 68,5 | 11,721 | 7,322 | 0,1676 | 1,4636 | 193,9 | 5,020 |
| 4 | VEH | 1757 | 51,8 | 35,142 | 1203 | 15,669 | 7,238 | 0,0288 | 11,695 | 46,9 | 0,102 |
| 5 | VEH | 1126 | 49,7 | 17,620 | 161,2 | 15,216 | 5,656 | 0,03 | 3,3876 | 9575 | 0,0732 |
| 6 | VEH | 1344 | 27,749 | 17,236 | 101,1 | 4,169 | 2,3484 | 0 | 1,3456 | 11631 | 0,098 |
| 7 | VEH | 2154 | 51,9 | 85,6 | 25,067 | 1,5148 | 2,1768 | 0,0276 | 0,3264 | 13805 | 5,577 |
| 8 | VEH | 3402 | 566 | 134,7 | 56,4 | 31,195 | 9,856 | 0,592 | 3,1556 | 39828 | 5,766 |
| 9 | VEH | 1713 | 89,2 | 38,771 | 262,0 | 19,002 | 9,127 | 0,0316 | 6,140 | 13158 | 0,3408 |
| 10 | KET 3 | 968 | 40,3 | 14,549 | 6,303 | 0,844 | 0,2844 | 0,0292 | 0,056 | 20854 | 2,648 |
| 11 | KET 3 | 1108 | 65,7 | 19,923 | 7,256 | 0,8684 | 0,4204 | 0,0268 | 0,0688 | 16448 | 1,6428 |
| 12 | KET 3 | 6058 | 293,8 | 94,8 | 136,3 | 16,327 | 5,571 | 0,0344 | 2,6736 | 27785 | 0,6032 |
| 13 | KET 3 | 2280 | 88,9 | 28,560 | 41,8 | 5,037 | 1,6196 | 0,0228 | 0,364 | 25142 | 1,2708 |
| 14 | KET 3 | 3319 | 57,3 | 31,055 | 314,8 | 6,739 | 2,9612 | 0,0216 | 2,7628 | 19033 | 0,1024 |
| 15 | KET 3 | 4839 | 511 | 105,6 | 28,878 | 8,268 | 1,714 | 0,07 | 1,2316 | 36068 | 4,514 |
| 16 | KET 3 | 828 | 85,6 | 15,086 | 7,696 | 3,3348 | 0,6812 | 0,0392 | 0,2572 | 18269 | 1,688 |
| 17 | KET 3 | 1419 | 113,9 | 26,718 | 12,643 | 2,7756 | 0,9556 | 0,0368 | 0,1796 | 27492 | 0,884 |
| 18 | KET 3 | 3394 | 62,4 | 58,0 | 23,894 | 0,8512 | 0,754 | 0,0244 | 0,148 | 21382 | 5,501 |
| 19 | KET 3 | 2240 | 92,5 | 26,334 | 33,019 | 4,586 | 1,6628 | 0,02 | 0,2868 | 26141 | 0,9864 |

| Sample No | Treat-ment | MCA(a) | MCA(b) | MCA(o) | TCA | TCDCA | TDCA | TLCA | TMCA(a+b) | TUDCA | UDCA |
|---|---|---|---|---|---|---|---|---|---|---|---|
| 1 | VEH | 372,2 | 403 | 25,525 | 629 | 57,4 | 27,305 | 0,2768 | 1995 | 17,906 | 16,200 |
| 2 | VEH | 914 | 1115 | 224,9 | 1283 | 143,4 | 75,5 | 2,3044 | 3811 | 39,750 | 38,646 |
| 3 | VEH | 979 | 330,1 | 42,2 | 97,1 | 8,875 | 2,6332 | 1,0004 | 587 | 1,1128 | 17,762 |
| 4 | VEH | 182,4 | 256,1 | 6,814 | 8257 | 1397 | 437 | 4,384 | 13495 | 336,9 | 16,005 |
| 5 | VEH | 135,9 | 165,5 | 9,296 | 4360 | 681 | 176,6 | 2,722 | 5413 | 166,1 | 13,088 |
| 6 | VEH | 164,1 | 183,5 | 9,463 | 2791 | 135,0 | 68,4 | 0,9028 | 2907 | 61,0 | 9,166 |
| 7 | VEH | 343,3 | 259,8 | 33,357 | 113,8 | 7,782 | 6,800 | 0,6832 | 303,8 | 1,382 | 20,762 |
| 8 | VEH | 900 | 396,9 | 58,1 | 41,0 | 8,854 | 1,5908 | 1,3696 | 348,9 | 1,7952 | 88,2 |
| 9 | VEH | 259,4 | 316,1 | 25,806 | 9656 | 1340 | 388,9 | 5,263 | 8051 | 442 | 28,904 |
| 10 | KET 3 | 137,8 | 124,5 | 23,289 | 5,138 | 0,56 | 0,1032 | 0,1132 | 111,8 | 0,1692 | 12,448 |
| 11 | KET 3 | 202,2 | 133,0 | 22,685 | 6,431 | 0,9012 | 0,2436 | 0,0816 | 85,8 | 0,3832 | 13,216 |
| 12 | KET 3 | 930 | 739 | 94,6 | 1679 | 273,6 | 77,1 | 3,0044 | 2379 | 59,1 | 69,9 |
| 13 | KET 3 | 391,5 | 261,9 | 44,3 | 623 | 89,7 | 21,195 | 1,2616 | 1042 | 19,568 | 22,083 |
| 14 | KET 3 | 325,6 | 560 | 41,9 | 5582 | 269,2 | 98,2 | 1,0108 | 5003 | 145,7 | 31,249 |
| 15 | KET 3 | 1009 | 926 | 181,9 | 221,8 | 58,4 | 8,868 | 7,145 | 1342 | 10,591 | 107,0 |
| 16 | KET 3 | 268,4 | 213,7 | 30,001 | 35,853 | 13,896 | 1,9208 | 0,5924 | 297,0 | 2,3388 | 15,390 |
| 17 | KET 3 | 334,3 | 202,5 | 28,860 | 28,424 | 5,241 | 0,8032 | 0,2204 | 243,4 | 0,866 | 22,254 |
| 18 | KET 3 | 493 | 338,5 | 54,4 | 91,7 | 3,6844 | 1,6504 | 0,2192 | 360,7 | 1,2048 | 43,1 |
| 19 | KET 3 | 452 | 334,5 | 40,3 | 145,6 | 22,830 | 5,846 | 0,5752 | 791 | 6,102 | 24,257 |
