# Supplementary material for: The Nonsteroidal Anti-Inflammatory Drug Ketorolac Alters the Small Intestinal Microbiota and Bile Acids Without Inducing Intestinal Damage or Delaying Peristalsis in the Rat
Source: Front Pharmacol. 2021 Jun 4;12:664177. doi: 10.3389/fphar.2021.664177 (PMC8213092; doi:10.3389/fphar.2021.664177)

Supplementary Figure S1

The nonsteroidal anti-inflammatory drug ketorolac alters the small intestinal microbiota and bile acids without inducing intestinal damage or delaying peristalsis in the rat

Barbara Hutka, Bernadette Lázár, András S. Tóth, Bence Ágg, Szilvia B. László, Nóra Makra, Balázs Ligeti, Bálint Scheich, Kornél Király, Mahmoud Al-Khrasani, Dóra Szabó, Péter Ferdinandy, Klára Gyires, Zoltán S. Zádori*

*** Correspondence:**
Dr. Zoltán S. Zádori
zadori.zoltan@med.semmelweis-univ.hu

Figure S1. Heat map of Spearman's correlation coefficients between the small intestinal proportion of individual bile acids and the relative abundance of bacterial families. Individual p values were corrected by the false discovery rate method according to Benjamini and Hochberg, asterisks indicate q < 0.05.

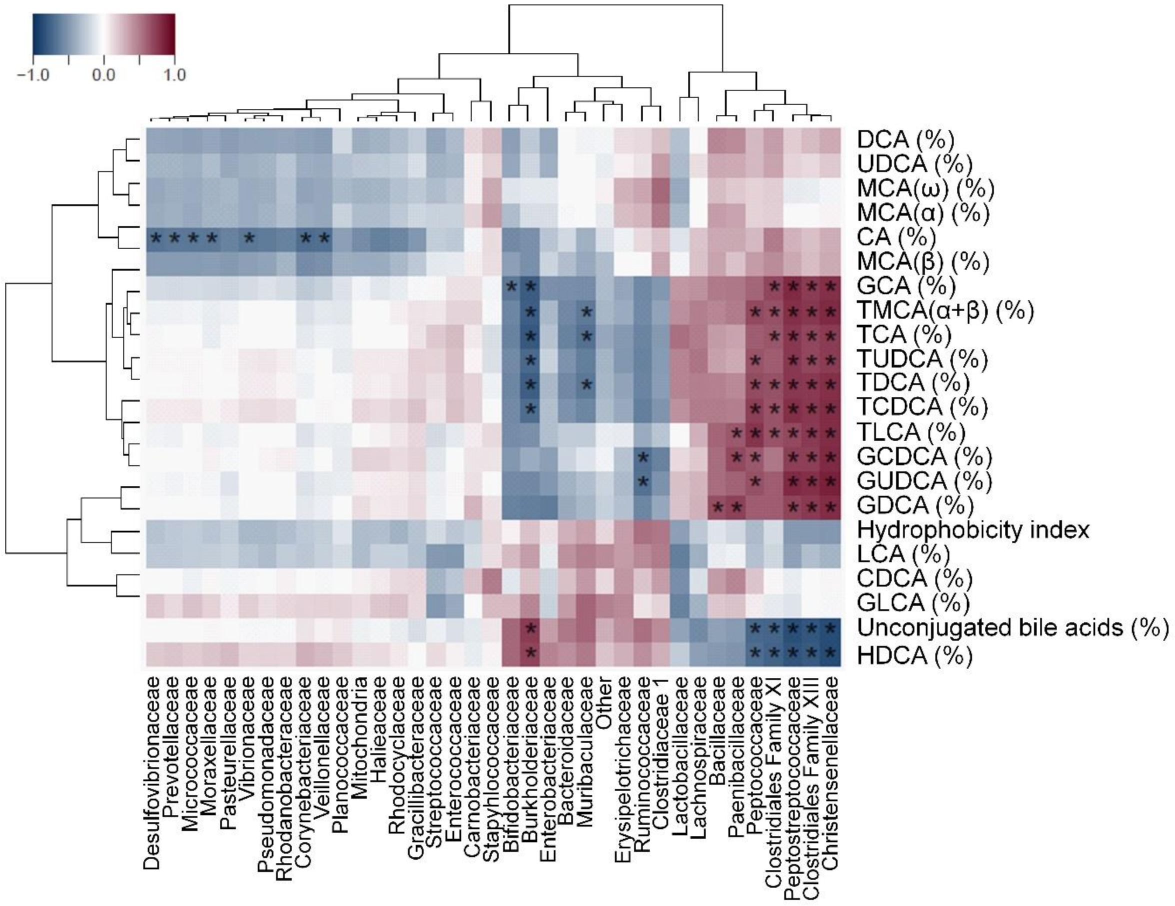

Supplement: Supplementary file 2 [file Image1.pdf]
